# Supplementary material for: Polyketide synthase gene clusters in the ericoid mycorrhizal fungus Oidiodendron maius: genome-wide prediction, expression profiling, and functional characterization of knockout mutants
Source: Front Plant Sci. 2026 Sep 16;17:1924430. doi: 10.3389/fpls.2026.1924430 (PMC13623903; doi:10.3389/fpls.2026.1924430)
Supplement: Supplementary file 1 [file SupplementaryFile1.zip › Supplementary Materials/Supplementary Folder 1.zip!/Oidma1_AssemblyScaffolds_Repeatmasked/knownclusterblast/cluster51/jgi.p_Oidma1_46252_mibig_hits.html]

| MIBiG Protein | Description | MIBiG Cluster | MiBiG Product | % ID | % Coverage | BLAST Score | E-value |
| --- | --- | --- | --- | --- | --- | --- | --- |
| BAC20567.1 | HMG-CoA\_reductase | BGC0000039 | Compactin\_biosynthetic\_gene\_cluster | 55 | 103.394255875 | 391 | 1.1e-108 |
| ABA02245.1 | HMG-CoA\_reductase | BGC0000098 | Monacolin\_K\_biosynthetic\_gene\_cluster | 52 | 104.960835509 | 385 | 6.2e-107 |
| AAD34556.1 | hydroxymethylglutaryl-coenzyme\_A\_reductase | BGC0000089 | Lovastatin\_biosynthetic\_gene\_cluster | 52 | 104.177545692 | 378 | 7.6e-105 |
| CBF85179.1 | conserved\_hypothetical\_protein | BGC0000673 | Ent-pimara-8(14),15-diene\_biosynthetic\_gene\_cluster | 52 | 102.349869452 | 349 | 6.4e-96 |
| BAD86804.1 | 3-hydroxy-3-methylglutaryl-CoA\_reductase | BGC0000286 | Viguiepinol\_biosynthetic\_gene\_cluster | 33 | 88.2506527415 | 142 | 9.1e-34 |
| CAX48660.1 | putative\_3-hydroxy-3-methylglutaryl-CoA\_reductase | BGC0001080 | Phenazine\_biosynthetic\_gene\_cluster | 34 | 88.2506527415 | 141 | 2.7e-33 |
| BAE78981.1 | 3-hydroxy-3-methylglutaryl-CoA\_reductase | BGC0001078 | Furaquinocin\_A\_biosynthetic\_gene\_cluster | 33 | 88.2506527415 | 140 | 3.5e-33 |
| ABS50444.1 | NapT2 | BGC0001079 | Napyradiomycin\_biosynthetic\_gene\_cluster | 34 | 88.2506527415 | 139 | 1e-32 |
| AHW81465.1 | EpaF | BGC0000934 | Endophenazines\_biosynthetic\_gene\_cluster | 33 | 91.3838120104 | 134 | 1.9e-31 |
| AGH68890.1 | HMG-CoA\_reductase | BGC0001083 | Merochlorin\_biosynthetic\_gene\_cluster | 34 | 82.7676240209 | 134 | 1.9e-31 |
| EWM63039.1 | hydroxymethylglutaryl-coenzyme\_A\_reductase | BGC0000679 | Diazepinomicin\_biosynthetic\_gene\_cluster | 33 | 88.2506527415 | 129 | 6.1e-30 |
| AEC13082.1 | 3-hydroxy-3-methylglutaryl\_coenzyme\_A\_reductase | BGC0000060 | Fostriecin\_biosynthetic\_gene\_cluster | 30 | 83.8120104439 | 100 | 4e-21 |
